# A novel strategy for protein production using non-classical secretion pathway in *Bacillus subtilis*

**DOI:** 10.1186/s12934-016-0469-8

**Published:** 2016-04-28

**Authors:** Jingqi Chen, Liuqun Zhao, Gang Fu, Wenjuan Zhou, Yuanxia Sun, Ping Zheng, Jibin Sun, Dawei Zhang

**Affiliations:** Tianjin Institute of Industrial Biotechnology, Chinese Academy of Sciences, Tianjin, 300308 People’s Republic of China; Key Laboratory of Systems Microbial Biotechnology, Chinese Academy of Sciences, Tianjin, 300308 People’s Republic of China; National Engineering Laboratory for Industrial Enzymes, Tianjin, 300308 People’s Republic of China

**Keywords:** *Bacillus subtilis*, d-Psicose 3-epimerase, Fusions, Non-classical secretion pathway, Localization

## Abstract

**Background:**

The Gram-positive bacterium *Bacillus subtilis* has been widely used as a cell factory for the production of proteins due to its generally regarded as safe (GRAS) nature and secretion capability. Of the known secretory pathways in *B. subtilis*, the majority of proteins are exported from the cytoplasm by Sec pathway, Tat pathway and ABC transporters, etc. However, the production of heterologous proteins by *B. subtilis* is unfortunately not that straight forward because of the bottlenecks in classical secretion pathways. The aim of this work is to explore a new method for protein production based on non-classical secretion pathway.

**Results:**

One d-psicose 3-epimerase (RDPE) which converts d-fructose into d-psicose from *Ruminococcus sp*. 5_1_39BFAA was successfully and substantially secreted into the extracellular milieu without the direction of signal peptide. Subsequently, we demonstrated that RDPE contained no native signal peptide, and the secretion of RDPE was not dependent on Sec or Tat pathway or due to cell lysis, which indicated that RDPE is a non-classically secreted protein. Then, we attempted to evaluate the possibility of using RDPE as a signal to export eighteen reporter proteins into the culture medium. Five of eleven homologous proteins, two of five heterologous proteins from other bacterium and two heterologous proteins of eukaryotic source were successfully secreted into the extracellular milieu at different secretion levels when they were fused to RDPE mediated by a flexible 21-bp linker to keep a distance between two single proteins. Furthermore, the secretion rates of two fusion proteins (RDPE-DnaK and RDPE-RFP) reached more than 50 %. In addition, most of the fusion proteins retained enzyme or biological activity of their corresponding target proteins, and all of the fusions still had the activity of RDPE.

**Conclusions:**

We found and identified a heterologous non-classically secreted protein RDPE, and showed that RDPE could direct proteins of various types into the culture medium, and thus non-classical protein secretion pathway can be used as a novel secretion pathway for recombinant proteins. This novel strategy for recombinant protein production is helpful to make *B. subtilis* as a more ideal cell factory for protein production.

**Electronic supplementary material:**

The online version of this article (doi:10.1186/s12934-016-0469-8) contains supplementary material, which is available to authorized users.

## Background

The production processes of recombinant proteins in microbial hosts are a major factor in modern biotechnology and bio based economies. Many organisms have the ability to secrete some native proteins into the culture medium at high concentrations. Thus, considerable effort has been aimed at developing an efficient secretion system for the production of recombinant proteins. Secretory expression offers many advantages when compared with cytoplasmic expression: it simplifies the detection and purification of the product, reduces the complexity of the bioprocess, minimises the cell-associated proteolytic degradation and improves the protein folding and quality [1].

Members of the genus *Bacillus* are prodigious producers of industrial enzymes, such as proteases and α-amylases, which are secreted across their single membrane system directly into the culture medium. In biotechnological processes for protein production, *Bacillus subtilis* has become most popular due to the complete lack of toxic by-products [2, 3], high product yields (20–25 g/L) [4], no pronounced codon bias [5] and excellent fermentation properties, etc. Importantly, the early sequencing of the *B. subtilis* genome represented an enormous technology push [6, 7], which was followed up by genome-wide gene function analysis studies, resulting into high amenability for genetic engineering [8]. Based on proteomics analysis, *B. subtilis* has the potential to export approximately 300 proteins [9]. Of the identified extracellular proteins 84 that are completely transported across the cytoplasmic membrane are synthesized with an amino-terminal signal peptide most of which should be translocated via the general secretion (Sec) pathway in an unfolded conformation [10]. Fewer proteins (like PhoD and YwbN) are released into the medium via the cleavable twin-arginine translocation (Tat) system in a folded conformation [11]. Still other proteins are exported into the medium via ATP-binding cassette (ABC) transporters [12]. With the development of investigation involved in Sec and Tat pathway, many Sec-dependent [13–15] and Tat-dependent [16, 17] signal peptides were applied in secretion of proteins of interest. However, the high-level secretion of heterologous proteins with signal peptides is unfortunately not that straight forward. Furthermore, every step in the process of Sec or Tat pathway involves dozens of translocation components that can be the source of the bottlenecks that cause reduced yields, which greatly limits the application of *B. subtilis* in proteins production on a wide scale.

Though many proteins that are secreted contain known secretion signals, proteins that are considered to be cytoplasmic proteins without any known signals or secretion motifs can also be found in the extracellular space. These proteins are termed as non-classically secreted proteins because their secretion route is still unclear [18]. As one of the most comprehensively studied Gram-positive bacteria, *B. subtilis* is also capable of secreting proteins via one or more non-classical secretion pathways. Haike et al. identified 17 typical cytoplasmic proteins that contain no known signal peptide [10]. Similarly, Tjalsma et al. listed 24 proteins found in the extracellular environment without having classical signal peptides and suggested that signal peptide independent protein secretion in bacteria is perhaps more common than previously thought [9]. Vitikainen et al. discovered a number of seemingly non-classically secreted proteins in *B. subtilis* through a structure–function analysis of the foldase protein (PrsA) [19]. Proteins involved in metabolism of amino acids, RocA and RocF, were initially found by Antelman et al. [20] to be non-classically secreted, but only RocF was later identified by Vitikainen et al. [19]. The vegetative catalase KatA that was previously considered to be an intracellular enzyme due to the absence of a signal peptide, was later found to be localized extracellularly when *B. subtilis* was grown in the rich medium (about 56 % of the total KatA) [21]. Of course, the detection of non-classically secreted proteins in the extracellular environment could obviously be attributed to cell lysis. However, Yang et al. have confirmed that the secretion of a heterologous protein Est55 and several cytoplasmic proteins without signal peptides in *B. subtilis* is a general phenomenon and is not a consequence of cell lysis. Furthermore, numerous researches of different groups in several bacterial species supported the fact that non-classically secreted proteins are, indeed, exported from the intact cells. Though the mechanisms of non-classical secretion are unidentified, non-classical secretion system has been applied in protein production. The non-secreted disordered nucleoskeletal-like protein (Nsp) was successfully exported when fused to non-classically secreted proteins [17]. Recently, Wang et al. used four non-classically secreted proteins to direct the export of Nsp, two of them to guide the secretion of alkaline phosphatase (PhoA), and one of them to lead the secretion of the thermostable β-galactosidase BgaB [22]. Though these examples indicate that the production of recombinant proteins can be achieved by non-classical secretion pathway, nearly all the yields of secreted proteins were very low. Therefore, more new non-classically secreted proteins need to be discovered and characterized for their application in the improvement of protein production in *B. subtilis*.

In this study, one d-psicose 3-epimerase (RDPE) which converts d-fructose into d-psicose from *Ruminococcus sp*. 5_1_39BFAA was successfully expressed and further secreted into the extracellular milieu without the direction of signal peptide, and was proved to be a non-classically secreted protein in *B. subtilis*. Subsequently, we evaluated the ability of RDPE to act as a signal to export recombinant proteins into the culture medium. Eighteen reporter proteins which were screened out from different sources were fused to RDPE linked by a flexible 21-bp linker to keep a distance between two single proteins. When fused to RDPE, five of eleven homologous proteins were secreted into the culture medium. Moreover, the non-classically secreted protein RDPE can direct the secretion of two of five heterologous proteins from other bacterium. Especially, two proteins from eukaryotes were both exported into the culture medium with the aid of RDPE. Importantly, the rates of the secreted portions of two fusion proteins (RDPE-DnaK and RDPE-RFP) were visibly more than 50 %. In addition, most of the fusion proteins retained their corresponding enzyme or biological activity, and all of the fusions still had the activity of RDPE. These results indicate that recombinant proteins can be exported into the medium with the direction of the non-classically secreted protein RDPE via non-classical secretion pathway, which is a novel strategy for protein production using this new potential secretion pathway.

## Results

### Heterologous expression of recombinant RDPE in *B. subtilis*

*Bacillus subtilis* naturally secretes large amounts of proteins directly into the culture medium, and most of secreted proteins usually contain typical signal peptides. Therefore, when we produce various proteins, the recombinant proteins of interest are usually equipped with effective Sec-dependent or Tat-dependent signal peptides with the aim of obtaining high-level secretion in *B. subtilis*. However, during our study of the enzyme RDPE, from *Ruminococcus sp*. 5_1_39BFAA [23], we found one interesting phenomenon that the recombinant RDPE can be exported into the medium without any signal peptides in *B. subtilis*.

**Fig. 1:**
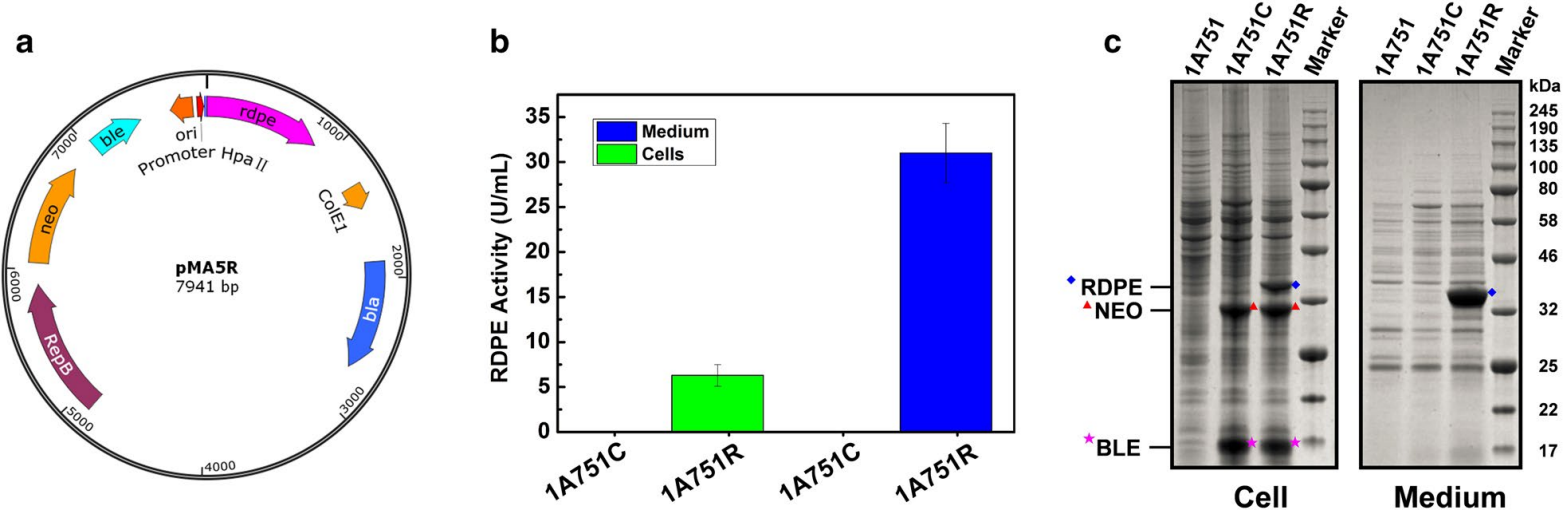
Expression and secretion of recombinant RDPE in *B. subtilis*. **a** Vector map of the recombinant expression plasmid pMA5R. P_*HpaII*_, a widely used promoter from *Staphylococcus aureus*; RBS, ribosome binding site; ColE1, origin of replication for *E. coli*; *bla*, ampicillin resistance; RepB, origin of replication for *B. subtilis*; *neo*, kanamycin resistance. *rdpe*, the gene encoding RDPE. **b** Enzyme activity of recombinant RDPE in medium and cell fraction with 48 h incubation. Data represent the mean of three parallel experiments, and *error bars* represent standard error. **c** SDS-PAGE analysis of expression of recombinant RDPE in medium and cell fractions by *B. subtilis* 1A751R at incubation of 48 h. 1A751 and 1A751C are regarded as the negative controls

*B. subtilis* 1A751 was transformed with the recombinant expression plasmid pMA5R (Fig. 1a), which contains the gene *rdpe* encoding RDPE, resulting into the recombinant strain 1A751R. With 48 h cultivation, the intracellular and extracellular RDPE activity reached 6.3 and 31.0 U/mL respectively (Fig. 1b). The results indicated that about 83 % (obtained by activity comparison) of the total recombinant RDPE was secreted into the culture medium. SDS-PAGE analysis (Fig. 1c) showed that the target band in the culture medium was significantly broader than that in cells, which is consistent with the activity analysis. Subsequently, by prediction of signal peptide using the online software SignalP 4.1 (http://www.cbs.dtu.dk/services/SignalP/), we found that RDPE doesn’t contain its native signal peptide sequence (Additional file 1: Fig. S1). However, studies on *B. subtilis* protein secretion have shown that the majority of secretory proteins contain classical signal peptides that direct them into the extracellular environment via Sec or Tat pathway [12, 24]. Thus, we speculated that RDPE might be a heterologous non-classically secreted protein in *B. subtilis*.

### Secretion of RDPE via non-classical secretion pathway

**Fig. 2:**
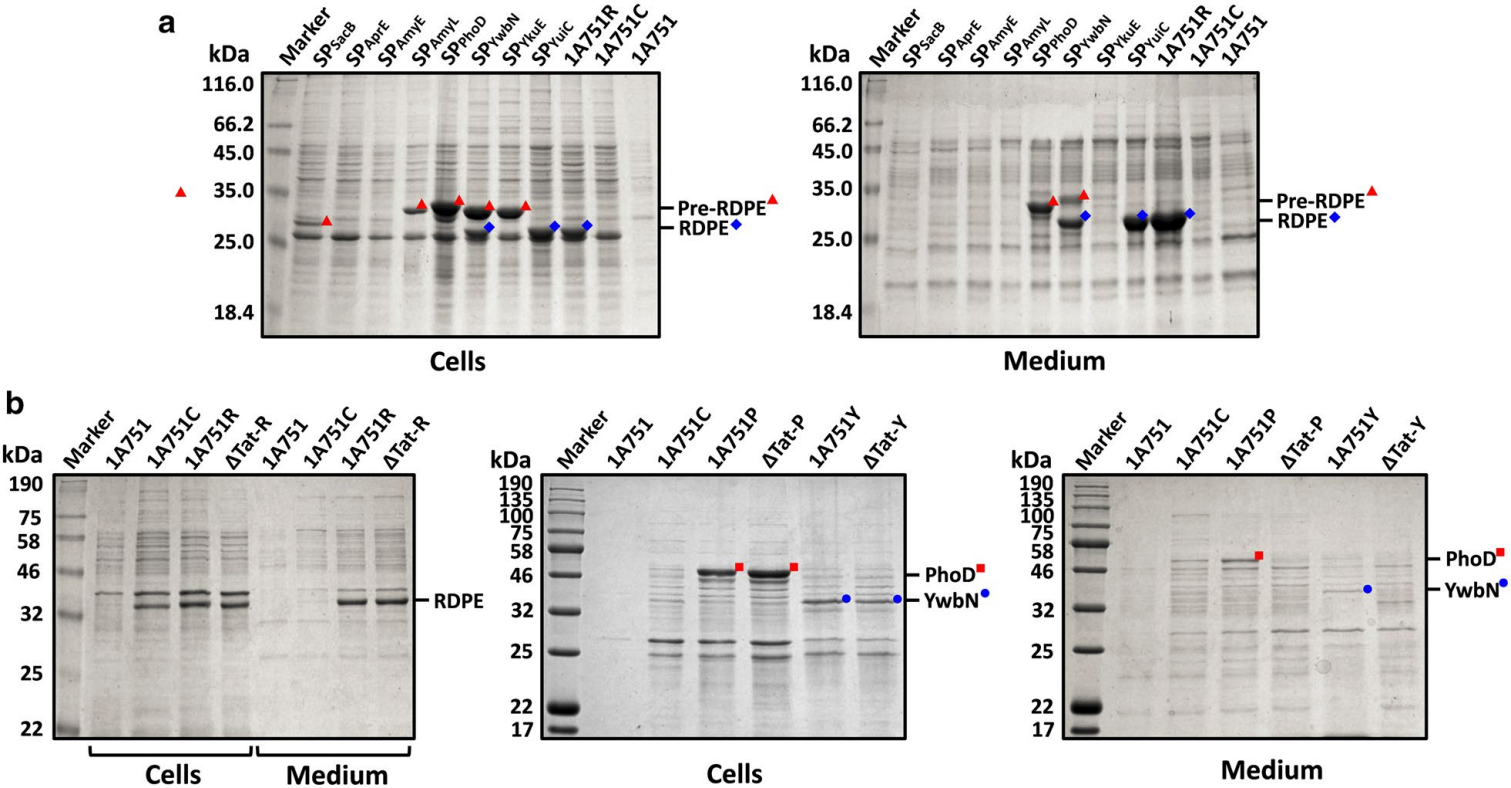
RDPE is secreted independently on Sec or Tat pathway. **a** Expression of RDPE fused to Sec or Tat signal peptides in *B. subtilis* 1A751. 1A751 and 1A751C (1A751 containing pMA5) were regarded as negative controls. 1A751R, 1A751 containing pMA5R (encoding RDPE). **b** Secretion of RDPE in the strain with deficiency of Tat pathway. 1A751P, 1A751 containing pMA5P (encoding PhoD). 1A751Y, 1A751 containing pMA5Y (encoding YwbN). ΔTat-R, 1A751T3R (encoding RDPE). ΔTat-P, 1A751T3P (encoding PhoD). ΔTat-Y, 1A751T3Y (encoding YwbN)

To confirm that RDPE is secreted via non-classical secretion pathway in *B. subtilis*, we must exclude that its secretion is Sec- or Tat-dependent. Because Sec pathway is essential for *B. subtilis*, the deficiency of Sec components could not be studied [25]. Therefore, we fused RDPE to four Sec-type signal peptides (SP_SacB_, SP_AprE_, SP_AmyL_ and SP_AmyE_) and the constructed plasmids were transformed into 1A751. SP_SacB_, SP_AprE_ and SP_AmyE_ are signal peptides from *B. subtilis*, and SP_AmyL_ is an efficient signal peptide from *Bacillus licheniformis* [26]. With the fusion of SP_SacB_ and SP_AmyL_ respectively, two RDPE precursors (SP_SacB_-RDPE and SP_AmyL_-RDPE) were detected in the cells, but no mature RDPE was exported into the medium. With the fusion of SP_AprE_ and SP_AmyE_ respectively, no RDPE (pre- or mature) was expressed in the cells or secreted into the medium, which might be caused by rapidly degradation due to incorrected fold (Fig. 2a). Moreover, we also ever expressed RDPE in a series of strains with single or combinational overexpression of Sec components (SecA, SRP, SecYEG, Ftsy, SecDF and YwbN, etc.) [26]; however, no improvement of RDPE secretion level was obtained (data not shown). Based on the above observations, we conclude that RDPE secretion is independent on Sec pathway in *B. subtilis*.

Our pilot studies have shown that RDPE with the Tat-type signal peptide SP_PhoD_ from *B. subtilis* was successfully exported into the growth medium (unpublished). We thus hypothesized that RDPE secretion might be related to Tat pathway, which directs folded proteins across the bacterial membranes [24]. To test this hypothesis, we firstly fused RDPE to different Tat-type signal peptides (SP_YwbN_, SP_YkuE_ and SP_YuiC_) from *B. subtilis*. The obtained recombinant plasmids were transformed into 1A751. In the cell fraction, pre-RDPE was detected when SP_PhoD_, SP_YwbN_ or SP_YkuE_ were fused to RDPE, and mature RDPE was detected when SP_YwbN_ or SP_YuiC_ were fused RDPE, respectively. In the medium, pre-RDPE was secreted with the fusion of SP_PhoD_ or SP_YwbN_, and mature RDPE was exported with the fusion of SP_YwbN_ or SP_YuiC_ (Fig. 2a). From the above results, it seemed like that RDPE could be secreted into the medium with the direction of Tat-dependent signal peptides SP_PhoD_, SP_YwbN_ or SP_YuiC_, although not all signal peptides were cleaved out. However, previous studies have shown that a heterologous cytoplasmic protein GFP fused to Tat signal peptides can be exported into the medium not through Tat-pathway [27, 28]. Thus, we next generated a mutant strain with deficiency of Tat pathway. From SDS-PAGE analysis (Fig. 2b), PhoD and YwbN which are strictly dependent on Tat pathway were not detected extracellularly in the mutant strain ΔTat compared with that in the parental strain 1A751, confirming that Tat pathway had been blocked thoroughly. However, RDPE without any signal peptides still could be successfully exported into the medium in ΔTat, and the extracellular level of RDPE was nearly same with that in the parental strain 1A751, which indicated that the knockout of Tat pathway had no effect on RDPE secretion. Based on all the results, we conclude that RDPE secretion is not Tat-dependent in *B. subtilis* though RDPE can be exported when fused to some Tat-dependent signal peptides.

Although it has been confirmed that neither Sec nor Tat system is involved in the secretion of RDPE, the release of RDPE is possibly due to cell lysis. In fact, except for *rdpe* encoding RDPE, the recombinant expression plasmid pMA5R also contains the genes *neo* and *ble* encoding kanamycin nucleotidyltransferase (NEO) and bleomycin resistance protein (BLE) from *Staphylococcus aureus* respectively. As shown in Fig. 1c, both NEO (29.2 kDa) and BLE (15.2 kDa) were successfully expressed in the cells, but neither NEO nor BLE was detected in the medium. Therefore, it can be excluded that the secretion of RDPE is due to cell lysis. From all the above descriptions, we can conclude that RDPE is exported across the cytoplasmic membrane into the growth medium via an unidentified secretion pathway, namely, non-classical secretion pathway.

### Localization of RDPE fusions to homologous proteins in *B. subtilis*

The previous review on protein secretion in *B. subtilis* lists seventeen typical cytoplasmic proteins identified in the extracellular milieu that contain no known signal peptide [10]. Wang et al. explored the possibility of using four of these non-classically secreted proteins as signals to export recombinant proteins [22]. Though partial fusion proteins were successfully secreted, the secretion levels of proteins were too low which could just be detected by western blotting. Based on the new non-classically secreted protein RDPE and its high secretion level, we therefore attempted to use recombinant RDPE as a signal to export recombinant proteins into the medium.

**Fig. 3:**
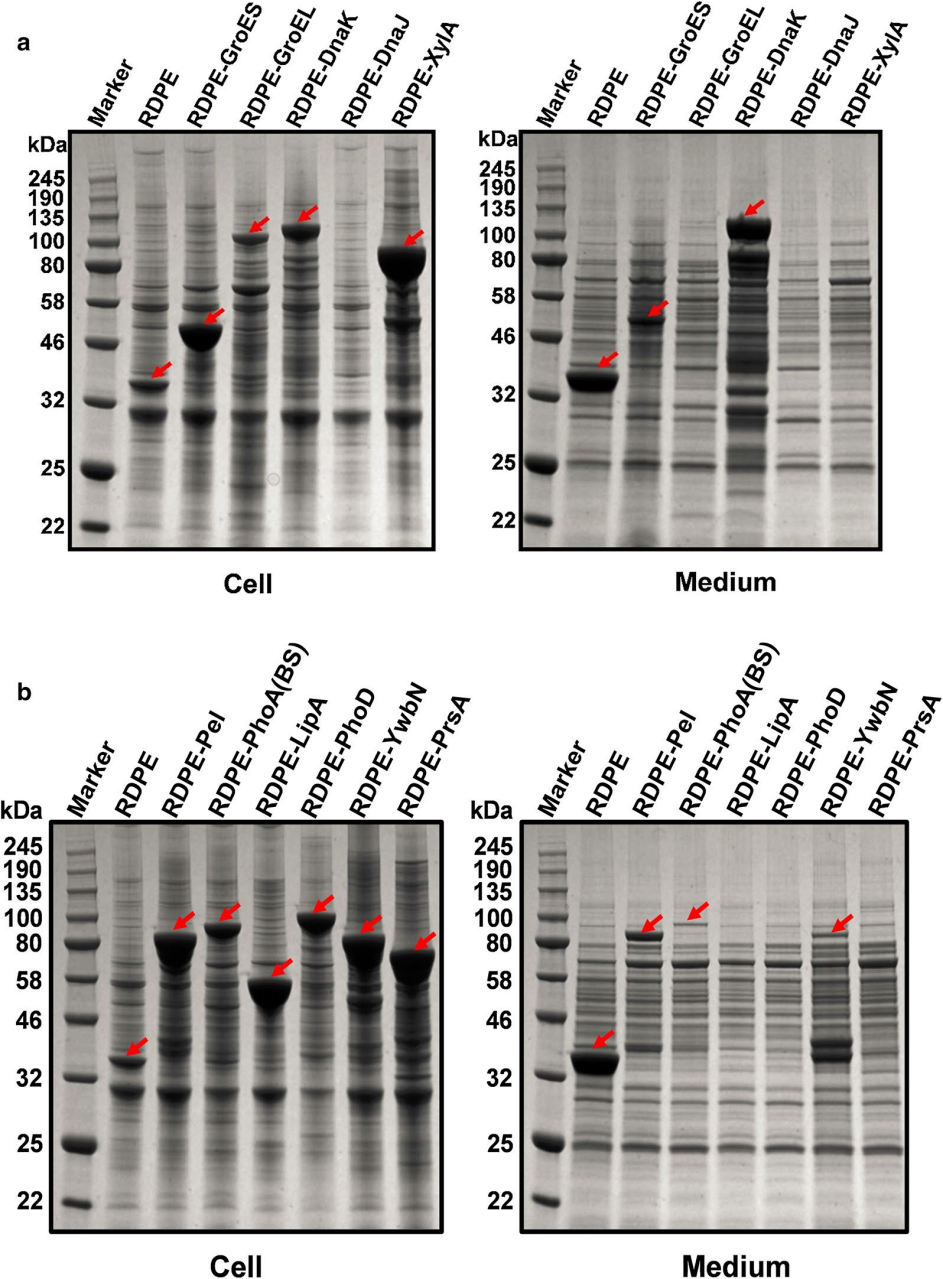
The expression and secretion of fusion proteins in *B. subtilis*. **a** SDS-PAGE analysis of expression of five cytoplasmic proteins from *B. subtilis* fused to RDPE in medium and cell fractions. **b** SDS-PAGE analysis of expression of five secreted proteins and one membrane protein from *B. subtilis* fused to RDPE in medium and cell fractions. RDPE represents 1A751C as the control

Firstly, we chose five cytoplasmic proteins GroES, GroEL, DnaK, DnaJ and XylA from *B. subtilis* as the reporter proteins. GroES, GroEL, DnaK and DnaJ are intracellular molecular chaperones which can act either independently or synergistically in a consecutive manner to facilitate the folding and assembly of certain proteins [3]. XylA was xylose isomerase from *B. subtilis* which can convert xylose to xylulose [29]. These five proteins were fused to RDPE linked by a 21-bp flexible DNA linker, respectively. The achieved plasmids encoding RDPE-GroES, RDPE-GroEL, RDPE-DnaK, RDPE-DnaJ and RDPE-XylA fusions were successfully transferred into *B. subtilis* 1A751. As shown in Fig. 3a, all fusions RDPE-GroES, RDPE-GroEL, RDPE-DnaK and RDPE-XylA except RDPE-DnaJ were detected in the cytoplasm by SDS-PAGE analysis, and the intracellular expression levels of these four fusions, especially RDPE-GroES and RDPE-XylA, were rather substantial. RDPE-GroES and RDPE-DnaK fusions were further detected in the supernatant, and the extracellular expression level of RDPE-DnaK was much higher than the intracellular expression level. Then, five naturally secreted proteins (Pel, PhoA (BS), LipA, PhoD and YwbN) and one membrane protein PrsA were fused to RDPE with the same strategy as above. The enzymes Pel, PhoA (BS) and LipA are Sec-dependent proteins in *B. subtilis* [30, 31]. PhoD and YwbN are strictly Tat-dependent proteins in *B. subtilis* [11]. Before we fused these four secreted proteins to RDPE, all of their native signal peptides were removed to avoid effecting the secretion of corresponding fusion proteins. PrsA is a lipoprotein that consists of a 33-kDa lysine-rich protein part and the N-terminal cysteine with a thiol-linked diacylglycerol anchoring the protein to the outer leaflet of the cytoplasmic membrane [32, 33]. The recombinant plasmids encoding RDPE-Pel, RDPE-PhoA (BS), RDPE-LipA, RDPE-PhoD, RDPE-YwbN and RDPE-PrsA were transferred into *B. subtilis* 1A751. All these six fusions were successfully and substantially expressed in cytoplasm (Fig. 3b). Of the six fusion proteins, three (RDPE-Pel, RDPE-PhoA (BS) and RDPE-YwbN) were detected in the culture medium by SDS-PAGE analysis.

In summary, ten of eleven fusions were successfully and largely expressed in the cells, and five of ten expressed fusions were detected in the medium. Different from these five extracellular fusion proteins, another five fusions (RDPE-GroEL, RDPE-XylA, RDPE-LipA, RDPE-PhoD and RDPE-PrsA) appeared just in the cell fraction, which also suggests that the appearance of the fused proteins in the extracellular milieu was not due to cell lysis. By comparison of the target bands in SDS-PAGE analysis, the intracellular and extracellular sizes of secreted fusion proteins were nearly identical. In addition, we also determined the enzyme activity of the fusion proteins. All the ten expressed fusions can convert d-fructose to d-psicose, suggesting that the fusions retained the activity of RDPE. The secreted fusions RDPE-Pel and RDPE-PhoA (BS) still maintained Pel and PhoA activity respectively (Table 1). The intracellular RDPE-LipA had no lipase activity, which is because of that intracellular LipA usually maintains unfolded state. Based on the results above, we can conclude that the non-classically secreted protein RDPE is able to lead the secretion of proteins (though not all) into the extracellular milieu.

**Table 1:** The enzyme activity of fusion proteins

Name	Intracellular activity (U/mL)	Extracellular activity (U/mL)
pMA5	–	–
RDPE	7.3 ± 0.8	32 ± 1.7
RDPE-XylA	1830 ± 45	–
RDPE-Pel	–	180 ± 13
RDPE-PhoA(BS)	–	145 ± 18
RDPE-LipA	–	–

The enzyme activity of fusion proteins refers to the enzyme activity of the corresponding target proteins

The results represent data from three independent experiments

– Not detected

### Localization of RDPE fusions to heterologous proteins from other bacterium

From the above results, we can see that about half of native proteins could be exported into the culture medium with the aid of non-classically secreted protein RDPE in *B. subtilis*. Because all of these reporter proteins are homologous proteins from *B. subtilis*, we therefore chose several proteins from other bacterium as the reporter proteins to further study the possibility of using non-classically secreted proteins to lead the secretion of recombinant proteins.

**Fig. 4:**
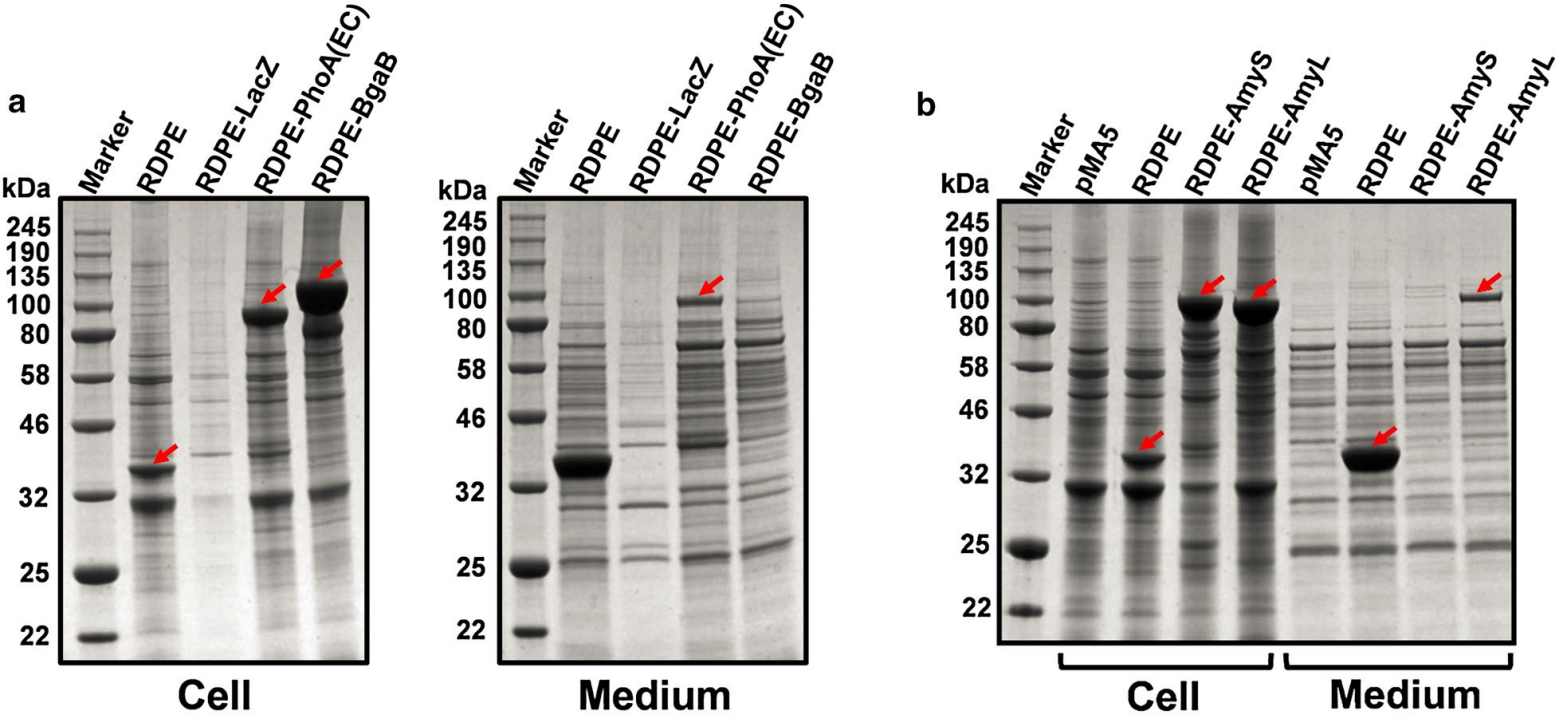
The expression and secretion of fusion proteins in *B. subtilis*. **a** SDS-PAGE analysis of expression of fusion proteins RDPE-LacZ, RDPE-PhoA(EC) and RDPE-BgaB in medium and cell fractions. **b** SDS-PAGE analysis of expression of fusion proteins RDPE-AmyS and RDPE-AmyL in medium and cell fractions. pMA5 represents 1A751C as the negative control

Five candidate proteins (LacZ, PhoA (EC), BgaB, AmyS and AmyL) were screened out. LacZ and PhoA (EC) are cytoplasmic and secreted enzymes from *Escherichia coli* respectively. BgaB and AmyS are intracellular and extracellular enzymes in *Geobacillus stearothermophilus* respectively. AmyL is secreted α-amylase from *Bacillus licheniformis*. Similarly, this five proteins were fused to non-classically secreted protein RDPE, resulting into five fused proteins (RDPE-LacZ, RDPE-PhoA (EC), RDPE-BgaB, RDPE-AmyS and RDPE-AmyL), respectively. With the previous strategy, the signal peptides of PhoA (EC), AmyS and AmyL had been deleted. Then, the corresponding activity of the fusion proteins was measured (Table 2), and the expression levels of the fusions were checked by SDS-PAGE analysis (Fig. 4). The fusion RDPE-LacZ was not detected whether intracellularly or extracellularly for some unknown reasons. Four fusions (RDPE-PhoA (EC), RDPE-BgaB, RDPE-AmyS and RDPE-AmyL) were detected in the cytoplasm. Two (RDPE-PhoA (EC) and RDPE-AmyL) of these four fusions were obviously detected extracellularly by SDS-PAGE, and their respective enzyme activity reached about 870 and 63 U/mL at 48 h cultivation. Despite substantial expression of the fusions RDPE-PhoA (EC), RDPE-AmyS and RDPE-AmyL in cytoplasm, the corresponding enzyme (PhoA, AmyS and AmyL) activities were not detected. This is because the correct folding of PhoA (EC), AmyS and AmyL occurs only when they are secreted into the extracellular milieu. This location-specific folding property of these enzymes have led to their wide use as reporters of protein localization in prokaryotic cells. In short, RDPE cannot direct the export of BgaB and AmyS, but PhoA (EC) and AmyL were both successfully secreted under the direction of RDPE via non-classical secretion pathway.

**Table 2:** The enzyme activity of fusion proteins

Name	Intracellular activity (U/mL)	Extracellular activity (U/mL)
pMA5	0	0
RDPE	7.3 ± 0.8	32 ± 1.7
RDPE-LacZ	–	–
RDPE-PhoA(EC)	–	870 ± 27
RDPE-BgaB	18 ± 1.2	0.08 ± 0.03
RDPE-AmyS	–	–
RDPE-AmyL	–	63 ± 11

The enzyme activity of fusion proteins refers to the enzyme activity of the corresponding target proteins

The results represent data from three independent experiments

– Not detected

### Localization of RDPE fusions to heterologous proteins from eukaryotes

**Fig. 5:**
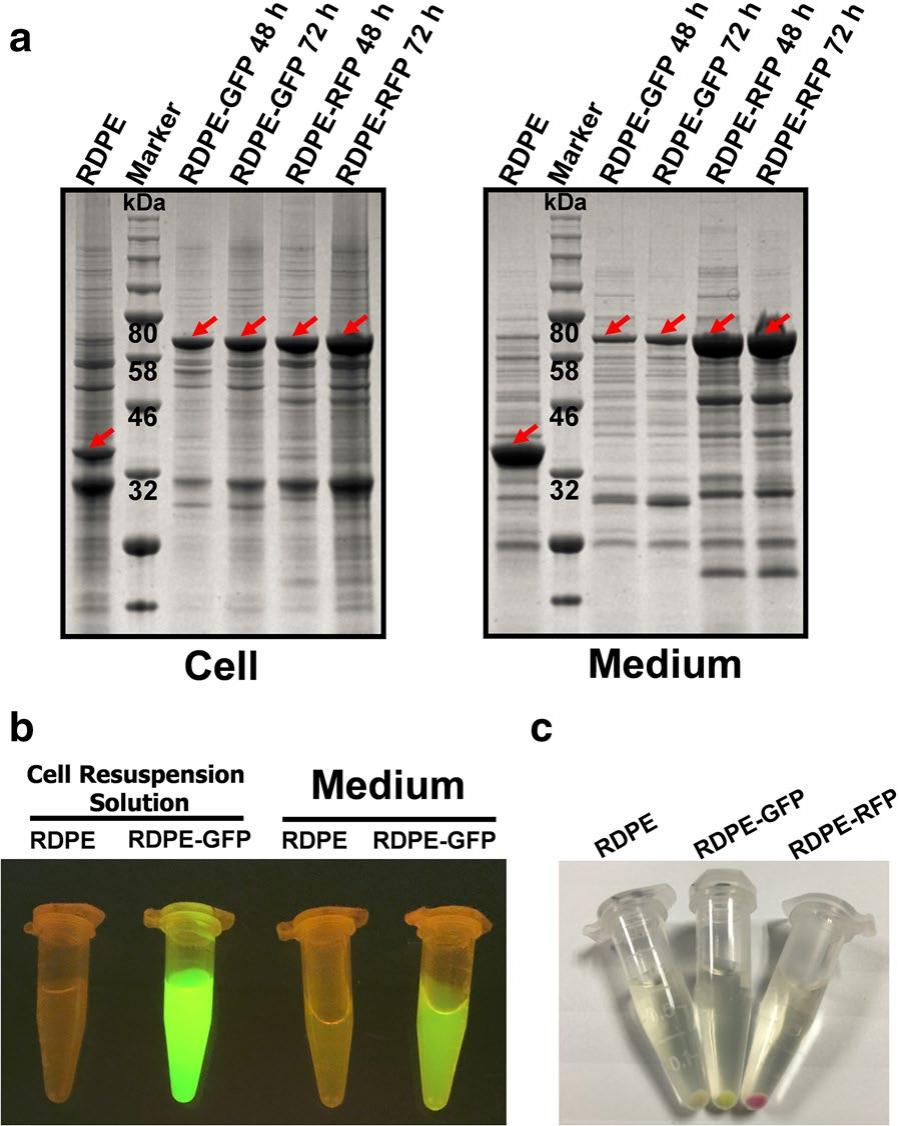
The expression and secretion of fusion proteins in *B. subtilis*. **a** SDS-PAGE analysis of expression of fusion proteins RDPE-GFP and RDPE-RFP in medium and cell fractions. **b** The excitation of fluorescence of the fusion RDPE-GFP under blue light. **c** Observation of the color of collected cells

All of the chosen proteins above were from prokaryotes, a part of which were successfully exported into the culture medium with the direction of RDPE. To expand application range, therefore, we screened out two model proteins from eukaryotes as reporter proteins to further study the capacity of RDPE to export recombinant proteins. Green fluorescent protein (GFP) from *Aequorea victoria* [34] and red fluorescence protein (RFP) from *Discosoma coral* [35] are frequently used as reporter proteins which are usually cytoplasmic. In this study, non-classically secreted protein RDPE was tested for use as a signal to lead the secretion of GFP and RFP. The plasmids encoding RDPE-GFP and RDPE-RFP fusions were successfully transformed into the expression strain *B. subtilis* 1A751. As shown in Fig. 5a, both RDPE-GFP and RDPE-RFP were detected intracellularly and extracellularly by SDS-PAGE analysis at 48 and 72 h, respectively. In particular, RDPE-RFP expression level in the extracellular milieu was significantly higher than that in cytoplasm. However, the extracellular RDPE-GFP secretion level was less than the intracellular RDPE-GFP expression level. Meanwhile, the biological activity of the fusion RDPE-GFP was further determined (Fig. 5b). With the excitation by blue light, irradiated cell resuspension solution and culture medium both emitted green fluorescence. In addition, we can clearly see that the collected RDPE-GFP and RDPE-RFP cells looked green and red, respectively, compared with RDPE cells (Fig. 5c). These observations were consistent with the analysis of relative fluorescence unit (RFU) (Table 3), which suggests that the fusion proteins RDPE-GFP and RDPE-RFP still retained biological activity. With the aid of RDPE, approximately 22 % of RDPE-GFP and 69 % of RDPE-RFP (RFU comparison) were exported into the culture medium. These results show that non-classically secreted protein RDPE can lead the secretion of GFP and RFP via non-classical secretion pathway.

**Table 3:** The fluorescence (RFU) of fusion proteins

Strains	RDPE	RDPE-GFP	RDPE-RFP
Cell resuspension solution	0	2210 ± 130	3430 ± 96
Medium	0	640 ± 54	7600 ± 147

The results represent data from three independent experiments

– Not detected

### Cleavage of RDPE from target proteins

**Fig. 6:**
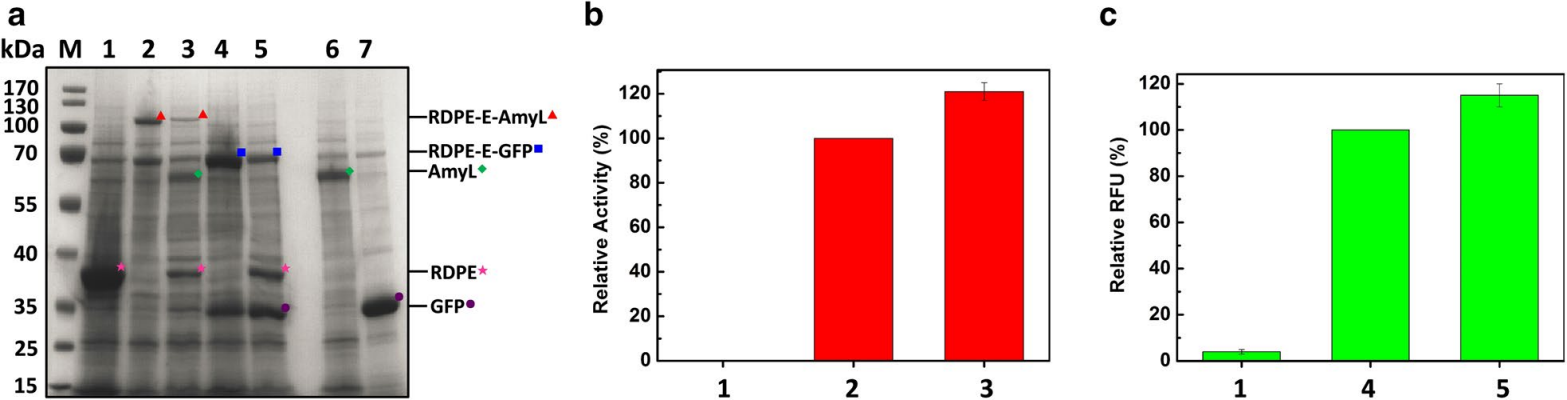
Cleavage of fusion proteins by enterokinase. The *B. subtilis* 1A751 cells harboring pMA5R16E or pMA5R17E were cultured in SR medium in flasks at 37 °C. The culture medium was concentrated, and twofold concentrate was obtained. 20 mL concentrate was reacted with the enterokinase. **a** SDS-PAGE analysis. *Lane 1*, the concentrate containing RDPE; *Lane 2*, the concentrate containing RDPE-E-AmyL; RDPE-E-AmyL contains the enterokinase cleavage site between RDPE and AmyL; *Lane 3*, the concentrate containing RDPE-E-AmyL following cleavage with the enterokinase; *Lane 4*, the concentrate containing RDPE-E-GFP; RDPE-E-GFP contains the enterokinase cleavage site between RDPE and GFP; *Lane 5*, the concentrate containing RDPE-E-GFP following cleavage with the enterokinase; *Lane 6*, the concentrate containing AmyL; *Lane 7*, the concentrate containing GFP. **b** The relative activity analysis. *Column 1* is the control. *Column 2* is served as 100 %. **c** The relative RFU (relative fluorescence units) analysis. *Column* 1 is the control. *Column 2* is served as 100 %. The samples 1, 2, 3, 4 and 5 in **b** or **c** are same with that in **a**. Data represent the mean of three parallel experiments, and *error bars* represent standard error

In order to clear whether fusion of two proteins will compromise the biological activity of target proteins, we introduced the enterokinase cleavage site between the RDPE and AmyL or RDPE and GFP, considering the convenience of determination of enzyme and biological activity. As shown in Fig. 6a, the fusion proteins RDPE-E-AmyL and RDPE-E-GFP were both efficiently cleaved after 16 h incubation with the enterokinase under the reaction condition studied. Then, we determined the corresponding enzyme and biological activity. The α-amylase activity of cleaved RDPE-E-AmyL was slightly higher than that of RDPE-E-AmyL (Fig. 6b). Similarly, the relative fluorescence unit of cleaved RDPE-E-GFP was also a bit higher than that of RDPE-E-GFP (Fig. 6c). The results suggest that the fusion of two proteins just slightly compromises the biological activity of the target proteins. In addition, the RDPE-E-AmyL and RDPE-E-GFP fusion proteins were excreted to the medium at levels comparable to RDPE-AmyL and RDPE-GFP, respectively, indicating that the introduction of the enterokinase cleavage site did not affect the excretion of fusion proteins.

## Discussion

The genome of *B. subtilis* 168 is 4215 kbp in length and contains about 4100 genes that are predicted to include over 250 extracellular proteins; the majority of these proteins are secreted through the known pathway [36, 37]. However, proteomic studies have revealed that genome-based predictions reflect only 50 % of the actual composition of the extracellular proteome. This significant discrepancy is mainly due to the difficulties in the prediction of extracellular proteins lacking signal peptides (including cytoplasmic proteins) and lipoproteins [36]. These findings suggest that, in addition to the well-known secretion pathways, *B. subtilis* can utilize some unknown mechanisms, such as non-classical secretion pathway, to release such signal-less proteins into the extracellular environment. In fact, exported proteins without signal peptides have been identified by several researchers in various microorganisms [38–43], and the list of proteins known to be released without signal peptides is steadily growing. Except for homologous proteins, some heterologous proteins also can be secreted via non-classical secretion pathway. For example, Yang et al. have proved that Est55, a carboxylesterase without a classical signal peptide from *G. stearothermophilus*, was exported into the medium via a non-classical secretion pathway.

In this study, when we attempted to express a heterologous protein, RDPE from *Ruminococcus sp*. 5_1_39BFAA, in *B. subtilis*, we found that the recombinant RDPE was successfully secreted into the medium without any additional signal peptides. It also doesn’t contain its own signal peptide or secretion motif. We therefore speculated that RDPE might be a non-classically secreted protein in *B. subtilis* like Est55 mentioned previously. To verify this speculation, we firstly confirmed that the secretion pathway of RDPE was neither Sec pathway nor Tat pathway by fusing Sec- or Tat- dependent signal peptides to RDPE and deleting Tat-related genes to block Tat pathway. Moreover, it was also ruled out that RDPE was exported into the extracellular milieu due to cell lysis. As a result, we can conclude that RDPE is one of non-classically secreted proteins which are located in the extracellular milieu despite the absence of known signal peptides or other targeting peptides [18]. Because the secretion mechanism of RDPE is unclear, its secretion pathway thus belongs to non-classical secretion pathway. In most cases, classical secretion pathways have been used to produce the recombinant proteins, however, less attention was paid to recombinant protein secretion using non-classical secretion pathway.

To produce the recombinant protein using non-classical secretion pathway, the non-classically secreted protein RDPE was tested to export eighteen various reporter proteins (Additional file 2: Table S1) into the extracellular milieu. According to source, these proteins fall into three categories: homologous proteins, heterologous proteins from other bacterium and heterologous proteins from eukaryotes. Firstly, eleven homologous proteins (five cytoplasmic proteins, five extracellular proteins and one membrane protein) were fused to RDPE to investigate the ability of non-classical secretion protein to act as a signal to export recombinant proteins into the culture medium. The fusion RDPE-DnaJ was not detected neither in cytoplasm nor in medium, which might be caused by degradation by intra- and extracellular proteases or some unknown reasons. Five of ten expressed proteins were able to be secreted at different yield levels with the aid of RDPE. Particularly, more than 50 % of the total RDPE-DnaK was transported into the extracellular milieu. The results suggest that RDPE can export partial homologous proteins across the cell membrane via unknown translocation mechanism. We noted that both cytoplasmic proteins (GroES and DnaK) and secreted proteins (PhoA and YwbN) without native signal peptides can be secreted by RDPE. Therefore, there might be no clear rule for homologous protein secretion via non-classical secretion pathway. In addition, all the expressed RDPE fusions had RDPE activity; the activity of Pel and PhoA (BS) is location specific, so only the secreted RDPE-Pel and RDPE-PhoA (BS) had Pel activity and PhoA (BS) activity, respectively. These results indicated that the fusion of two proteins didn’t inactivate these two enzymes. Then five heterologous proteins from other bacterium were employed as the reporter proteins to be fused to RDPE. The fusion RDPE-LacZ was not detected in cells or medium. The proteins PhoA(EC) from Gram-negative bacteria and AmyL from Gram-positive bacteria were able to be secreted with the direction of RDPE. Although the secretion efficiency was not very high, the results show that heterologous proteins from other bacterium can be exported into exterior led by RDPE, and the secretion of reporter proteins doesn’t depend on classification of bacterium. Similarly, all the expressed fusions retained RDPE activity. The secreted RDPE-PhoA (EC) and RDPE-AmyL both maintained respective reporter protein activity. At last, we attempted to use RDPE as the signal to export the heterologous proteins (GFP and RFP) from eukaryotes which cannot be secreted in bacterium. Fortunately, both GFP and RFP were exported into the extracellular milieu with the direction of RDPE. Importantly, the ratio of secreted fusion RDPE-RFP could reach about 69 %. The results indicate non-classically secreted protein RDPE can exported heterologous proteins from eukaryotes into the exterior of the cell. Meanwhile, the fusions RDPE-GFP and RDPE-RFP also kept RDPE activity and fluorescence activity. In this study, we tested eighteen reporter proteins fused to RDPE in total, two of which were not detected whether intracellularly or extracellularly because of some unknown reasons. All other proteins were expressed in cytoplasm, and nine of these sixteen expressed proteins were successfully exported across the cell membrane into the extracellular milieu. Considering the application of the strategy of producing recombinant proteins using non-classical secretion pathway, the cleavage of RDPE from the fusion proteins was performed. The results indicated that the action of fusion just slightly compromised the biological activity of target proteins, which suggests that the strategy of producing recombinant proteins using RDPE as a secretion signal is valuable. In conclusion, the non-classical protein secretion pathway can be exploited as a novel secretion pathway for recombinant proteins, and is an excellent complement to the classical secretion pathway.

Although the recombinant proteins can be secreted with the aid of RDPE via the non-classical secretion pathway, more unknown aspects of this new secretion pathway need to be further investigated detailedly. Firstly, we need to find out in detail the export mechanism of RDPE or the signal triggering its secretion, so that the RDPE or RDPE fusions secretion can be thoroughly improved by optimizing the secretion pathway or secretion signal. In this study, though we have demonstrated that the secretion pathway of RDPE is not Sec or Tat pathway and excluded the possibility that RDPE was exported due to cell lysis, no clear secretion principle or signal was found in the secretion process. Recently, Yang et al. showed that the internal hydrophobic helix of enolase is essential as a signal for secretion and the intact long N-terminus including the hydrophobic helix domain is required to serve as a non-cleavable signal for the secretion of enolase [44]. Martin et al. provided evidence for an important role of caspase-1 in unconventional protein secretion via secretome analysis using iTRAQ proteomics [45]. However, these findings just suggest some exploratory speculations, so the accurate secretion signal and system need to be identified in the future. Secondly, not all reporter proteins can be exported with the direction of the non-classical secretion protein RDPE, so the standard of reporter proteins which can be successfully secreted needs to be explored. The gene source, the native localization and the size of reporter proteins seem not to effect the secretion of reporter proteins. The reporter proteins from *B. subtilis* (GroES, DnaK, Pel, PhoA (BS) and YwbN), other bacterium (PhoA (EC) and AmyL) and eukaryotes (GFP and RFP) all can be successfully exported with the direction of RDPE. Cytoplasmic (GroES, DnaK, GFP and RFP) and secreted (Pel, PhoA (BS), YwbN, PhoA(EC) and AmyL) proteins also can be secreted with the aid of RDPE. The reporter proteins with different sizes (GroES (10 kDa), DnaK (67 kDa), Pel (44 kDa), PhoA(BS) (47 kDa), YwbN (41 kDa), PhoA (EC) (50 kDa), AmyL (53 kDa), GFP (26 kDa) and RFP (25 kDa)) can be transported across the cell membrane led by RDPE. From these above results, we speculate that there might be no correlation between the gene source, the native localization or the size of reporter proteins and the secretion of reporter proteins. In total, the recombinant target protein itself plays a vital role in protein secretion when using the non-classical secretion pathway. Thirdly, although nine of sixteen expressed proteins were successfully secreted with the direction of RDPE, the yields of most proteins were very low. In this study, the ratios of extracellular RDPE-DnaK and RDPE-RFP were much higher than that of intracellular RDPE-DnaK and RDPE-RFP. However, the extracellular levels of other proteins were lower than the intracellular levels. As the yield and efficiency of fusion proteins are both low, more research is needed to explore the favoured substrates in the non-classical pathway and suitable non-classically secreted proteins for the desired target proteins.

More investigations should be carried out to reveal various aspects of the new non-classical secretion pathway of RDPE, and more non-classical secretion proteins including the homologous and heterologous need to be discovered in future. This study developed a new strategy for recombinant protein production via non-classical secretion pathway, which has vast perspectives and great significance for theoretical research and industrial applications.

## Conclusions

We found and identified a new non-classically secreted protein RDPE, and thus developed a novel strategy for recombinant protein production via non-classical secretion pathway in *B. subtilis*. In this study, we successfully used the non-classically secreted protein RDPE as a signal to export nine proteins of various gene sources, native localizations and sizes into the extracellular milieu, the ratios of two of which in the culture medium were significantly higher than 50 %, suggesting that non-classical secretion pathway can be exploited as a novel secretion pathway for recombinant proteins in *B. subtilis*. However, more unknown aspects of the non-classical secretion pathway of RDPE need to be investigated detailedly and systematically, involving export mechanism, standard of reporter proteins and enhancement of the yield, etc. In addition, more homologous and heterologous non-classical secretion proteins need to be discovered. As a complement to the classical secretion pathway, the non-classical secretion pathway will make *B. subtilis* as a more ideal cell factory for protein secretion with continuous progress of relevant study.

## Methods

### Bacterial strains, plasmids and growth conditions

Bacterial strains and plasmids used in this study are listed in Tables 4, 5, respectively. The bacterial strains *B. licheniformis* CICC 10181 and *G. stearothermophilus* ATCC 31195 were used as the sources of the SP_AmyL_, AmyL (*amyl*) gene, AmyS (*amys*) gene and BgaB (*bgaB*) gene, respectively. *E. coli* DH5α served as a host for cloning and plasmid preparation. *B. subtilis* 1A751, which is deficient in two extracellular proteases (*nprE, aprE*), was used as a host for protein expression. The plasmid pMA5 is an *E. coli/B. subtilis* shuttle vector and used to clone and express protein. Transformants of *E. coli* and *B. subtilis* were selected on Luria–Bertani (LB) agar (1 % (w/v) peptone, 0.5 % (w/v) yeast extract, 1 % (w/v) NaCl and 2 % (w/v) agar), supplemented with ampicillin (100 μg/mL), spectinomycin (200 μg/mL), chloramphenicol (12.5 μg/mL) or kanamycin (50 μg/mL) depending on the plasmid antibiotic marker. *E. coli* DH5α was incubated in LB medium supplemented with ampicillin (100 μg/mL) at 37 °C. *B. subtilis* was cultivated in SR medium [1.5 % (w/v) peptone, 2.5 % (w/v) yeast extract and 0.3 % (w/v) K_2_HPO_4_, pH 7.2] contained additionally kanamycin (50 μg/mL) at 37 °C. All of the strains were incubated under a shaking condition at 200 rpm. All of the experiments were repeated at least 3 times and mean values were used for comparison.

**Table 4:** Strains used in this study

Strains	Genotype and/or relevant characteristic(s)	Source
*E. coli* DH5α	F^−^∆*lac*U169(Ø80d *lac*Z∆M15) *sup*E44 *hsd*R17 *rec*A1 *gyr*A96 *end*A1 *thi*-1 *rel*A1	Invitrogen
*B. subtilis* 168	*trpC2*	Lab stock
*B. subtilis* 1A751	*egl*S∆102 *bgl*T/*bgl*S∆EV *apr*E *npr*E *his*	BGSC
*B. licheniformis* CICC 10181	Wild-type *B. licheniformis*, *amyL* gene	CICC
*G. stearothermophilus* ATCC 31195	Wild-type *G. stearothermophilus, amyS* gene, *bgaB* gene	ATCC
1A751C	1A751 containing pMA5; Km^r^	This work
1A751R	1A751 containing pMA5R; Km^r^	This work
1A751RS1	1A751 containing pMA5RS1; Km^r^	This work
1A751RS2	1A751 containing pMA5RS2; Km^r^	This work
1A751RS3	1A751 containing pMA5RS3; Km^r^	This work
1A751RS4	1A751 containing pMA5RS4; Km^r^	This work
1A751RT1	1A751 containing pMA5RT1; Km^r^	Lab stock
1A751RT2	1A751 containing pMA5RT2; Km^r^	This work
1A751RT3	1A751 containing pMA5RT3; Km^r^	This work
1A751RT4	1A751 containing pMA5RT4; Km^r^	This work
1A751P	1A751 containing pMA5P; Km^r^	This work
1A751Y	1A751 containing pMA5Y; Km^r^	This work
1A751S	1A751∆*araR*::P*araR*-Spe; Spe^r^	Lab stock
1A751T1	1A751S ∆*tatAdCd*; Spe^r^	This work
1A751T2	1A751S∆*tatAdCd* ∆*tatAyCy*; Spe^r^	This work
1A751T3	1A751S∆*tatAdCd* ∆*tatAyCy* Δ*tatAc*; Spe^r^	This work
1A751T3P	1A751T3 containing pMA5P; Spe^r^ Km^r^	This work
1A751T3Y	1A751T3 containing pMA5Y; Spe^r^ Km^r^	This work
1A751T3R	1A751T3 containing pMA5R; Spe^r^ Km^r^	This work
1A751R1	1A751 containing pMA5R1; Km^r^	This work
1A751R2	1A751 containing pMA5R2; Km^r^	This work
1A751R3	1A751 containing pMA5R3; Km^r^	This work
1A751R4	1A751 containing pMA5R4; Km^r^	This work
1A751R5	1A751 containing pMA5R5; Km^r^	This work
1A751R6	1A751 containing pMA5R6; Km^r^	This work
1A751R7	1A751 containing pMA5R7; Km^r^	This work
1A751R8	1A751 containing pMA5R8; Km^r^	This work
1A751R9	1A751 containing pMA5R9; Km^r^	This work
1A751R10	1A751 containing pMA5R10; Km^r^	This work
1A751R11	1A751 containing pMA5R11; Km^r^	This work
1A751R12	1A751 containing pMA5R12; Km^r^	This work
1A751R13	1A751 containing pMA5R13; Km^r^	This work
1A751R14	1A751 containing pMA5R14; Km^r^	This work
1A751R15	1A751 containing pMA5R15; Km^r^	This work
1A751R16	1A751 containing pMA5R16; Km^r^	This work
1A751R17	1A751 containing pMA5R17; Km^r^	This work
1A751R18	1A751 containing pMA5R18; Km^r^	This work
1A751R16E	1A751 containing pMA5R16E; Km^r^	This work
1A751R17E	1A751 containing pMA5R17E; Km^r^	This work
1A751L	1A751 containing pMA5L; Km^r^	Lab stock
1A751G	1A751 containing pMA5G; Km^r^	Lab stock

*CICC* China Center of Industrial Culture Collection (http://www.chinacicc.org)

*ATCC* American Type Culture Collection

*BGSC* *Bacillus* Genetic Stock Center, USA

**Table 5:** Plasmids used in this study

Plasmids	Genotype and/or relevant characteristic(s)	Source
pDG	pDL derivative, Cm^r^; *gfp* gene	Lab stock
pDR	pDL derivative, Cm^r^; *rfp* gene	Lab stock
pMA5	*E. coli/B. subtilis* shuttle vector, P_*Hpa*II_; Ap^r^, Km^r^	Lab stock
pMA5R	pMA5 derivative, *rdpe*	This work
pMA5RS1	pMA5R derivative, SP_SacB_-*rdpe*	This work
pMA5RS2	pMA5R derivative, SP_AprE_-*rdpe*	This work
pMA5RS3	pMA5R derivative, SP_AmyE_-*rdpe*	This work
pMA5RS4	pMA5R derivative, SP_AmyL_-*rdpe*	This work
pMA5RT1	pMA5R derivative, SP_PhoD_-*rdpe*	Lab stock
pMA5RT2	pMA5R derivative, SP_YwbN_-*rdpe*	This work
pMA5RT3	pMA5R derivative, SP_YkuE_- *rdpe*	This work
pMA5RT4	pMA5R derivative, SP_YuiC_- *rdpe*	This work
pMA5P	pMA5 derivative, *phoD*	This work
pMA5Y	pMA5 derivative, *ywbN*	This work
pMA5R1	pMA5R derivative, *rdpe*-*groES*	This work
pMA5R2	pMA5R derivative, *rdpe*-*groEL*	This work
pMA5R3	pMA5R derivative, *rdpe*-*dnaK*	This work
pMA5R4	pMA5R derivative, *rdpe*-*dnaJ*	This work
pMA5R5	pMA5R derivative, *rdpe*-*xylA*	This work
pMA5R6	pMA5R derivative, *rdpe*-*pel*	This work
pMA5R7	pMA5R derivative, *rdpe*-*phoA(BS)*	This work
pMA5R8	pMA5R derivative, *rdpe*-*lipA*	This work
pMA5R9	pMA5R derivative, *rdpe*-*phoD*	This work
pMA5R10	pMA5R derivative, *rdpe*-*ywbN*	This work
pMA5R11	pMA5R derivative, *rdpe*-*prsA*	This work
pMA5R12	pMA5R derivative, *rdpe*-*lacZ*	This work
pMA5R13	pMA5R derivative, *rdpe*-*phoA(EC)*	This work
pMA5R14	pMA5R derivative, *rdpe*-*bgaB*	This work
pMA5R15	pMA5R derivative, *rdpe*-*amyS*	This work
pMA5R16	pMA5R derivative, *rdpe*-*amyL*	This work
pMA5R17	pMA5R derivative, *rdpe*-*gfp*	This work
pMA5R18	pMA5R derivative, *rdpe*-*rfp*	This work
pMA5R16E	pMA5R16 derivative, enterokinase cleavage site	This work
pMA5R17E	pMA5R17 derivative, enterokinase cleavage site	This work
pMA5L	pMA5 derivative, *amyl*	This work
pMA5R	pMA5 derivative, *gfp*	This work

### Primers and oligonucleotides

Polymerase chain reaction (PCR) primers and oligonucleotides used in this study were synthesized by GENEWIZ (Suzhou, China) and listed in Additional file 3: Table S2.

### General manipulation

PCRs were performed using PrimeSTAR Max DNA Polymerase (TaKaRa, Japan). DNA fragments and PCR products were excised from a 0.8 % agarose gel and purified by E.Z.N.A.^Tm^ Gel Extraction Kit (200) (Omega Bio-tek, Inc., USA) according to the manufactures’ instruction. E.Z.N.A.^Tm^ Plasmid Mini Kit I (Omega Bio-tek, Inc., USA) was applied for plasmid extraction according to the manufactures’ instruction. Genomic DNA isolation was carried out by TIANamp Bacteria DNA Kit (Tiangen Biotech (Beijing) Co., Ltd., China). All the DNA constructs were sequenced by GENEWIZ (Suzhou, China).

### Transformation of DNA

*Escherichia coli* transformation was carried out according to Sambrook et al. [46]. *B. subtilis* was naturally transformed using “Paris method” [47, 48].

### Construction of recombinant plasmids

Plasmids used in this study are listed in Table 5. All the recombinant plasmids were constructed by a sequence-independent “simple cloning” method without the need for restriction and ligation enzymes [49]. Based on the nucleotide sequence of *rdpe*, the primer pairs rdpe-F/rdpe-R were designed to amplify the fragment *rdpe* using the plasmid pET-RDPE as the template. The linear vector backbone was amplified using the primers pMA5-F and pMA5-R as the primers and the plasmid pMA5 as the template. rdpe-F/rdpe-R have the reverse complementary sequences of pMA5-F/pMA5-R, respectively. Then, the DNA multimer is generated based on these DNA templates by prolonged overlap extension PCR (POE-PCR). Eventually, the POE-PCR products (DNA multimer) were transformed into competent *E. coli* DH5α directly, yielding the recombinant plasmid pMA5R. Similarly, the signal peptide sequences SP_SacB_, SP_AprE_, SP_AmyE_, SP_AmyL_, SP_YwbN_, SP_YkuE_ and SP_YuiC_ were inserted into pMA5R upstream of the gene *rdpe*, resulting into the recombinant plasmids pMA5RS1, pMA5RS2, pMA5RS3, pMA5RS4, pMA5RT2, pMA5RT3 and pMA5RT4, respectively. With the same method, the plasmids pMA5P and pMA5Y were also constructed.

**Fig. 7:**
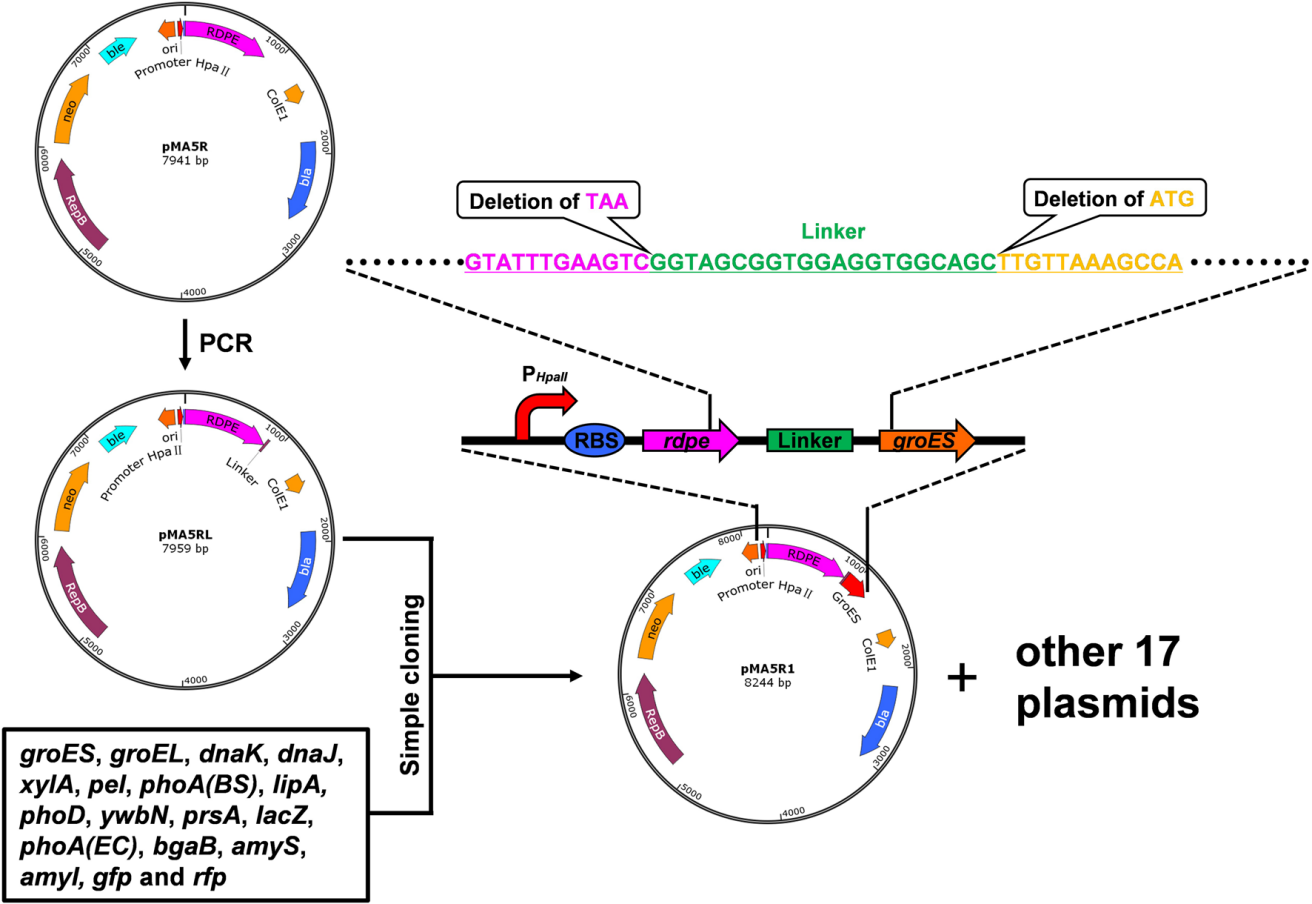
The construction of plasmids encoding fusion proteins used in this study. Eighteen fusion proteins were all under the control of the promoter P_*HpaII*_. A flexible 21-bp linker sequence was inserted between *rdpe* and the genes of reporter proteins respectively. The details of plasmid construction are described in “Methods” section

In order to fuse the reporter proteins to RDPE and avoid the negative interaction between the reporter proteins and RDPE, a flexible 21-bp linker was introduced between the nucleotide sequence of the reporter proteins and the nucleotide sequence of RDPE (Fig. 7). Therefore, the recombinant plasmid pMA5RL containing the 21-bp linker downstream of *rdpe* was firstly constructed. The linear vector pMA5R2 was cloned using the primers pMA5R-F2 and pMA5R-R2 containing 21-bp linker sequence at the 5′ terminus, and then ligated by T4 ligase, yielding the recombinant plasmid pMA5RL. Eighteen genes (*groES*, *groEL*, *dnaK*, *dnaJ*, *xylA*, *pel*, *phoA (BS)*, *lipA*, *phoD*, *ywbN*, *prsA*, *lacZ*, *phoA(EC)*, *bgaB*, *amyS*, *amyL*, *gfp* and *rfp*) were amplified using corresponding genomic DNA or plasmids as the templates. By the “simple cloning” method as described above, these fragments were then inserted into the plasmid pMA5RL, successively, downstream of the 21-bp linker, resulting into eighteen corresponding recombinant vectors.

In order to cleave RDPE from the target proteins, we introduce an enterokinase cleavage site between the RDPE and target proteins. The linear vectors pMA5R16E and pMA5R17E were cloned using the plasmids pMA5R16 and pMA5R17 as templates and pMA5R16E-F/R and pMA5R17E-F/R as primers, respectively. The primers pMA5R16E-F and pMA5R17E-F both contained the DNA sequence of the enterokinase cleavage site at the 5′ terminus. Then, the linear vectors were ligated by T4 ligase, yielding the recombinant plasmids pMA5R16E and pMA5R17E.

### Gene deletion

The method of marker-free gene deletion was as described by Liu et al. [50]. To obtain *B. subtilis* Δ*tatAdCd*, the fragment for deleting *tatAd*-*tatCd* operon was constructed as follows. The 0.9 kb *cat* (C) fragment was amplified from the pDG plasmid using the primers Cm1-F and Cm1-R. The 1.2 kb *araR* (R) fragment, containing the complete encoding region of the gene *araR*, was amplified from the *B. subtilis* 168 genome using the primers araR-F and araR-R. The UPAdCd (UP) and DNAdCd (DN), GAdCd (G) fragments were amplified from the *B. subtilis* 168 genome using the primers UP1-F and UP1-R, DN1-F and DN1-R, and G1-F and G1-R, respectively. Then, these five DNA fragments were fused into UP-DN-C-R-G by SOE-PCR using the primers UP1-F and G1-R. Eventually, the DNA fusion UP-DN-C-R-G were transformed into *B. subtilis* 1A751S, and *B. subtilis* Δ*tatAdCd* was obtained by homologous recombination between two homologous DN fragments. Similarly, the *tatAy*-*tatCy* operon was deleted and the strain Δ*tatAdCd*Δ*tatAyCy* was obtained. To delete the gene *tatAc*, the Δ*tatAc*::cat insertion deletion allet was generated by overlap extention PCR using primers UP3-F/UP3-R, DN3-F/DN3-R and Cm2-F/Cm2-R to amplify regions upstream and downstream of *tatAc* and a chloramphenicol resistance gene *cat* (pDG), respectively. At last, the strain ΔTat with complete deficiency of Tat pathway was constructed.

### Enterokinase cleavage of fusion proteins

To obtain fusion proteins, expression experiments were conducted with the *B. subtilis* 1A751 cells harboring corresponding plasmids in flask as described above. The resulting culture medium was concentrated using an Amicon1 Ultra-15 centrifugal filter unit with Ultracel-30 membrane (Millipore, Billerica, MA) and changed to the enterokinase reaction buffer (50 mM Tris–HCl, 2 mM CaCl_2_, pH 7.6). To cleave the fusion protein, 1 mL of enterokinase (New England Biolabs Catalog # P8070S) was added to 20 mL of the twofold concentrate, and kept at 25 °C for 16 h. The resulting reaction mixtures were subjected to SDS–PAGE analysis and activity analysis.

### Enzyme assays

The RDPE activity was analyzed by determining the amount of d-psicose obtained from d-fructose. One milliliter of reactions mixture contained d-fructose (20 g/L) in sodium phosphate buffer (50 mM, pH 8.0) and 200 μL fermentation broth. The reaction was incubated at 55 °C for 10 min, following by boiling at 100 °C for 10 min. The obtained d-psicose in the mixture was determined via high-performance HPLC system with a refractive index detector and a Sugar-PakTM Columnn (6.5 × 300 mm; Waters), which was eluted with ultrapure water at 80 °C and 0.4 mL/min. One unit of DPEase activity is defined as the amount of enzyme that catalyzed the production of 1 μmol d-psicose per minute. The activity of XylA, Pel, PhoA, LipA, LacZ, BgaB and AmyS (AmyL) were determined as described previously [26, 51–56].

### Fluorescence measurements

The fluorescence activity of RDPE-GFP and RDPE-RFP was monitored using the Multimode microplate reader (SpectraMax M5). The fermentation broth was centrifuged at 4000 rpm for 10 min, and then the cells and supernatant were obtained. Cells were diluted with equal volume of double distilled water. For RDPE-GFP, the extinction and emission wavelength were set at 488 and 523 nm, respectively. For RDPE-RFP, the extinction and emission wavelength were both 550 nm.

### SDS-PAGE analysis

Culture samples (1 mL) were harvested and the supernatant was separated from the culture medium by centrifugation (12,000*g*, 10 min, 4 °C). After adding 5× SDS-PAGE sample buffer, the supernatants were boiled for 10 min and proteins were separated in SDS-PAGE using the NuPAGE 10 % Bis–Tris Gel (Novex by Life Technologies, USA) in combination with MOPS SDS Running Buffer (Invitrogen Life Technologies, USA). PageRuler Prestained Protein Ladder (Invitrogen Life Technologies, USA) was used to determine the apparent molecular weight of separated proteins. Proteins were visualized with Coomassie Brilliant Blue.

## Additional files

10.1186/s12934-016-0469-8 Prediction of signal peptide by SignalIP 4.1.
10.1186/s12934-016-0469-8 Reporter proteins screened out for the study of non-classical secretion pathway.
10.1186/s12934-016-0469-8 Primers used in this study.

## Abbreviations

ABC transporters: ATP-binding cassette (ABC) transporters
RDPE: d-psicose 3-epimerase
GFP: green fluorescent protein
RFP: red fluorescence protein
POE-PCR: prolonged overlap extension PCR
