# Supplementary material for: A novel strategy for protein production using non-classical secretion pathway in Bacillus subtilis
Source: Microb Cell Fact. 2016 Apr 28;15:69. doi: 10.1186/s12934-016-0469-8 (PMC4850722; doi:10.1186/s12934-016-0469-8)
Supplement: Supplementary file 1 — 10.1186/s12934-016-0469-8 Prediction of signal peptide by SignalIP 4.1. [file 12934_2016_469_MOESM1_ESM.docx]

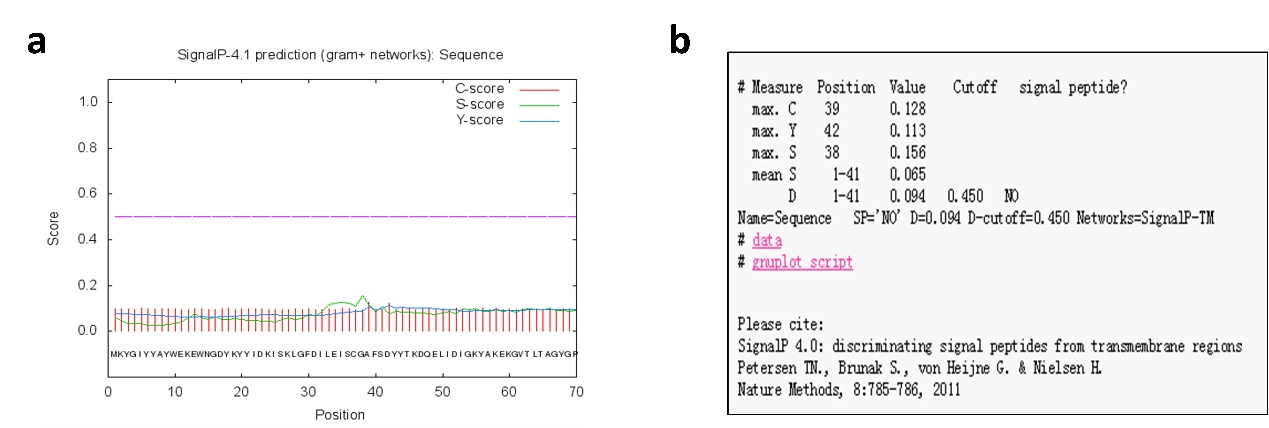


**Fig. S1 Prediction of signal peptide by SignalIP 4.1.** Signal peptide was predicted by SignalIP 4.1 (<http://www.cbs.dtu.dk/services/SignalP/>). By analysis of 291 amino acids using SignalIP 4.1, we can see RDPE doesn’t contain any typical signal peptides.
