# Supplementary material for: A novel strategy for protein production using non-classical secretion pathway in Bacillus subtilis
Source: Microb Cell Fact. 2016 Apr 28;15:69. doi: 10.1186/s12934-016-0469-8 (PMC4850722; doi:10.1186/s12934-016-0469-8)
Supplement: Supplementary file 2 — 10.1186/s12934-016-0469-8 Reporter proteins screened out for the study of non-classical secretion pathway. [file 12934_2016_469_MOESM2_ESM.docx]

**Table S1**

Reporter proteins screened out for the study of non-classical secretion pathway

| **NO.** | **Gene** | **Localisation** | **Function** | **Source** | **Molecular mass (kDa)** | **Gene length (bp)** | **Secretion pathway** |
| --- | --- | --- | --- | --- | --- | --- | --- |
| 1 | *groES* | Intracellular | Chaperone | *B. subtilis* | 10 | 285 |  |
| 2 | *groEL* | Intracellular | Chaperone | *B. subtilis* | 60 | 1635 |  |
| 3 | *dnaK* | Intracellular | Chaperone | *B. subtilis* | 67 | 1836 |  |
| 4 | *dnaJ* | Intracellular | Chaperone | *B. subtilis* | 41 | 1119 |  |
| 5 | *xylA* | Intracellular | Xylose isomerase | *B. subtilis* | 49 | 1338 |  |
| 6 | *pel* | Extracellular | Pectate lyase | *B. subtilis* | 44 | 1200 | Sec pathway |
| 7 | *phoA* | Extracellular | Alkaline phosphatase A | *B. subtilis* | 47 | 1293 | Sec pathway |
| 8 | *lipA* | Extracellular | Lipase | *B. subtilis* | 20 | 546 | Sec pathway |
| 9 | *phoD* | Extracellular | Phosphodiesterase | *B. subtilis* | 55 | 1503 | Tat pathway |
| 10 | *ywbN* | Extracellular | Unknown function | *B. subtilis* | 41 | 1119 | Tat pathway |
| 11 | *prsA* | Membrane | Chaperone | *B. subtilis* | 32 | 879 |  |
| 12 | *lacZ* | Intracellular | β-galactosidase | *E. coli* | 113 | 3075 |  |
| 13 | *phoA* | Extracellular | Alkaline phosphatase A | *E. coli* | 50 | 1353 | Sec pathway |
| 14 | *bgaB* | Intracellular | β-galactosidase | *G. stearothermophilus* | 74 | 2019 |  |
| 15 | *amyS* | Extracellular | α-amylase | *G. stearothermophilus* | 57 | 1548 | Sec pathway |
| 16 | *amyl* | Extracellular | α-amylase | *B. licheniformis* | 53 | 1452 | Sec pathway |
| 17 | *gfp* | Intracellular | Green fluorescent protein | *Aequorea victoria* | 26 | 717 |  |
| 18 | *rfp* | Intracellular | Red fluorescent protein | *Discosoma coral* | 25 | 678 |  |
