# Supplementary material for: A novel strategy for protein production using non-classical secretion pathway in Bacillus subtilis
Source: Microb Cell Fact. 2016 Apr 28;15:69. doi: 10.1186/s12934-016-0469-8 (PMC4850722; doi:10.1186/s12934-016-0469-8)
Supplement: Supplementary file 3 — 10.1186/s12934-016-0469-8 Primers used in this study. [file 12934_2016_469_MOESM3_ESM.docx]

**Table S2**

Primers used in this study

| Prmier | Sequence(5’-3’) |
| --- | --- |
| rdpe-F | TGCCACCTAAAAAGGAGCGATTTACATATGAAATATGGTATTTATTACGCTT |
| rdpe-R | GCTTGAGCTCGACTCTAGAGGATCCTTAGACTTCAAATACATGTTTTACA |
| PhoD-F | TGCCACCTAAAAAGGAGCGATTTACATATGGCATACGACAGTCGTTTTGATG |
| PhoD-R | GCTTGAGCTCGACTCTAGAGGATCCTCATCGGATTGCTTCACCCCGCCTTGG |
| YwbN-F | TGCCACCTAAAAAGGAGCGATTTACATATGAGCGATGAACAGAAAAAGCCAGAAC |
| YwbN-R | GCTTGAGCTCGACTCTAGAGGATCCTTATGATTCCAGCAAACGCTGGGCAATATA |
| pMA5-F | GGATCCTCTAGAGTCGAGCTCAAGCTAG |
| pMA5-R | ATGTAAATCGCTCCTTTTTAGGTGGCAC |
| pMA5R-F1 | AAATATGGTATTTATTACGCTTATTGGGAAAAGG |
| pMA5R-R1 | CATATGTAAATCGCTCCTTTTTAGGTGC |
| SPSacB-F | ACCTAAAAAGGAGCGATTTACATATGAACATCAAAAAGTTTGCAAAACAAGC |
| SPsacB-R | CAATAAGCGTAATAAATACCATATTTTTCTTTCGCAAACGCTTGAGTTG |
| SPAprE-F | ACCTAAAAAGGAGCGATTTACATATGAGAAGCAAAAAATTGTGGATCAGCT |
| SPAprE-R | CAATAAGCGTAATAAATACCATATTTAGCCTGCGCAGACATGTTGCT |
| SPAmyE-F | ACCTAAAAAGGAGCGATTTACATATGTTTGCAAAACGATTCAAAACCTCT |
| SPAmyE-R | CAATAAGCGTAATAAATACCATATTTAGCACTCGCAGCCGCCGGT |
| SPAmyL-F | ACCTAAAAAGGAGCGATTTACATATGAAACAACAAAAACGGCTTTACG |
| SPAmyL-R | CAATAAGCGTAATAAATACCATATTTCGCCGCTGCTGCAGAAT |
| SPYwbN-F | ACCTAAAAAGGAGCGATTTACATATGAGCGATGAACAGAAAAAGCCAG |
| SPYwbN-R | CAATAAGCGTAATAAATACCATATTTTGGCTTAGCCGCAGTCTGAAC |
| SPYukE-F | ACCTAAAAAGGAGCGATTTACATATGAAAAAGATGTCCAGAAGACAATTTC |
| SPYukE-R | CAATAAGCGTAATAAATACCATATTTGAGATACCTGGCATAGCCATATC |
| SPYuiC-F | ACCTAAAAAGGAGCGATTTACATATGATGTTGAATATGATCAGACGTTTGCT |
| SPYuiC-R | CAATAAGCGTAATAAATACCATATTTCAAGTCCTTCGCTTCAATTCCT |
| Cm1-F | CACTAACCTGCCCCGTTAGTTGAAGGCATTTTCTGTCAATGTTTTCTTAC |
| Cm1-R | GAACTGGAATGAGATTGAGCCATTTATTCATTCAGTTTTCGTGCGGACTGGCG |
| araR-F | TCTTCAACTAAAGCACCCATTAGTTC |
| araR-R | CTTCAACTAACGGGGCAGGTTAGTG |
| UP1-F | GCAGCATACCAGGCATACTAT |
| UP1-R | AGAATCAAGCCCGGTATTCC |
| DN1-F | ACATTGGAATACCGGGCTTGATTCTATTGATTACACCGCCTGATT |
| DN1-R | AACTAATGGGTGCTTTAGTTGAAGAGCCAAGCCATTCAAAAACAT |
| G1-F | GTCCGCACGAAAACTGAATGAATAACATCTTCGTCATCGCCATTATT |
| G1-R | ATGGACACGACAATCAGCAGA |
| UP2-F | GCACAGGTCAAAACGAAAGG |
| UP2-R | GGACCGAAGATAATCAGAGCA |
| DN2-F | TCGTTGCTCTGATTATCTTCGGTCCTCATGTTTTTGACAAGGCTCG |
| DN2-R | AACTAATGGGTGCTTTAGTTGAAGAGCGAGAACAAAACGGATATGG |
| G2-F | GTCCGCACGAAAACTGAATGAATAACAAAAAGCTGCCTGAATTGG |
| G2-R | GGATGACCGGCATTTGGAAC |
| UP3-F | ATTGTCACCGGGATCATCCT |
| UP3-R | AACTAATGGGTGCTTTAGTTGAAGAAAGCGCCGGCAGTTTATCAG |
| DN3-F | GTCCGCACGAAAACTGAATGAATAATAAACAAGCAACAAGCGGAC |
| DN3-R | TTTCAGATTCAGTCACCAAGGT |
| Cm2-F | TCTTCAACTAAAGCACCCATTAGTTC |
| Cm2-R | TTATTCATTCAGTTTTCGTGCGGAC |
| pMA5R-F2 | GGATCCTCTAGAGTCGAGCTCAAGC |
| pMA5R-R2 | GCTGCCACCTCCACCGCTACCGACTTCAAATACATGTTTTACAAAG |
| pMA5RL-F | GGATCCTCTAGAGTCGAGCTCAAGC |
| pMA5RL-R | GCTGCCACCTCCACCGCTACCGACTTC |
| groES-F | ATTTGAAGTCGGTAGCGGTGGAGGTGGCAGCTTGTTAAAGCCATTAGGTGATCGCGTTG |
| groES-R | CAAGCTAGCTTGAGCTCGACTCTAGAGGATCCTTAGCCGATAACAGCTAAAATGTCGC |
| groEL-F | TTTGAAGTCGGTAGCGGTGGAGGTGGCAGCATGGCAAAAGAAATTAAGTTTAGTGAAG |
| groEL-R | AGCTAGCTTGAGCTCGACTCTAGAGGATCCTTACATCATTCCACCCATACCGCCCATG |
| dnaK-F | ATTTGAAGTCGGTAGCGGTGGAGGTGGCAGCGTGAGTAAAGTTATCGGAATCGACTTAG |
| dnaK-R | AAGCTAGCTTGAGCTCGACTCTAGAGGATCCTTATTTTTTGTTTTGGTCGTCGTTTAC |
| dnaJ-F | TTTGAAGTCGGTAGCGGTGGAGGTGGCAGCATGAGTAAGCGTGATTACTATGAAGTGC |
| dnaJ-R | AAGCTAGCTTGAGCTCGACTCTAGAGGATCCTTAATCGCCTTTAAACGCGCGTTTTAC |
| xylA-F | ATTTGAAGTCGGTAGCGGTGGAGGTGGCAGCATGGCTCAATCTCATTCCAGTTCAATC |
| xylA-R | TAGCTTGAGCTCGACTCTAGAGGATCCTTATACTTCTAAAATGTATTGGTTCAATATCG |
| pel-F | GTATTTGAAGTCGGTAGCGGTGGAGGTGGCAGCGCTGATTTAGGCCACCAGACGTTGG |
| pel-R | CAAGCTAGCTTGAGCTCGACTCTAGAGGATCCTTAATTTAATTTACCCGCACCCGCTTG |
| phoA(BS)-F | GTATTTGAAGTCGGTAGCGGTGGAGGTGGCAGCGAGCTTCAGCAAACAGAAAAGGCCAG |
| phoA(BS)-R | CTAGCTTGAGCTCGACTCTAGAGGATCCTTATTTTCCAGTTTTTAAAATCTTAAATATG |
| lipA-F | GTATTTGAAGTCGGTAGCGGTGGAGGTGGCAGCGCTGAACACAATCCAGTCGTTATGG |
| lipA-R | AGCTAGCTTGAGCTCGACTCTAGAGGATCCTTAATTCGTATTCTGGCCCCCGCCGTTC |
| phoD-F | GTATTTGAAGTCGGTAGCGGTGGAGGTGGCAGCGCGCCTAACTTCTCAAGCTATCC |
| phoD-R | CAAGCTAGCTTGAGCTCGACTCTAGAGGATCCTCATCGGATTGCTTCACCCCGCCTTG |
| ywbN-F | TTTGAAGTCGGTAGCGGTGGAGGTGGCAGCGCTAAGCCATCGAAAAAGGATGAAAAAG |
| ywbN-R | CAAGCTAGCTTGAGCTCGACTCTAGAGGATCCTTATGATTCCAGCAAACGCTGGGCAA |
| prsA-F | ATTTGAAGTCGGTAGCGGTGGAGGTGGCAGCATGAAGAAAATCGCAATAGCAGCTATC |
| prsA-R | CTAGCTTGAGCTCGACTCTAGAGGATCCTTATTTAGAATTGCTTGAAGATGAAGAAGTG |
| lacZ-F | GTATTTGAAGTCGGTAGCGGTGGAGGTGGCAGCATGACCATGATTACGGATTCACTGG |
| lacZ-R | GCTAGCTTGAGCTCGACTCTAGAGGATCCTTATTTTTGACACCAGACCAACTGGTAATG |
| phoA(EC)-F | GTATTTGAAGTCGGTAGCGGTGGAGGTGGCAGCCGGACACCAGAAATGCCTGTTCTG |
| phoA(EC)-R | CAAGCTAGCTTGAGCTCGACTCTAGAGGATCCTTATTTCAGCCCCAGAGCGGCTTTC |
| bgaB-F | GTATTTGAAGTCGGTAGCGGTGGAGGTGGCAGCATGAATGTGTTATCCTCAATTTGTTACGGAG |
| bgaB-R | CAAGCTAGCTTGAGCTCGACTCTAGAGGATCCCTAAACCTTCCCGGCTTCATCATGC |
| amyS-F | GTATTTGAAGTCGGTAGCGGTGGAGGTGGCAGCGCCGCACCCTTTAACGGCACC |
| amyS-R | AGCTAGCTTGAGCTCGACTCTAGAGGATCCTTAAGGCCATGCCACCAGTCTAG |
| amyL-F | GTATTTGAAGTCGGTAGCGGTGGAGGTGGCAGCGCAAATCTTAATGGGACGCTGATGC |
| amyL-R | AGCTAGCTTGAGCTCGACTCTAGAGGATCCCTATCTTTGAACATAGATCGAAACCGAC |
| gfp-F | GAAGTCGGTAGCGGTGGAGGTGGCAGCATGAGTAAAGGAGAAGAACTTTTCACTGG |
| gfp-R | GAGCTCGACTCTAGAGGATCCCTATTTGTATAGTTCATCCATGCCATGTG |
| rfp-F | GTATTTGAAGTCGGTAGCGGTGGAGGTGGCAGCATGGCGAGTAGCGAAGACGTTATC |
| rfp-R | CTTGAGCTCGACTCTAGAGGATCCTTAAGCACCGGTGGAGTGACGACC |
| pMA5R16E-F | GCAAATCTTAATGGGACGCTGATGCAG |
| pMA5R16E-R | CTTGTCATCGTCATCGCTGCCACCTCCACCGCTACCGACTTCAAATACA |
| pMA5R17E-F | ATGAGTAAAGGAGAAGAACTTTTCACTG |
| pMA5R17E-F | CTTGTCATCGTCATCGCTGCCACCTCCACCGCTACCGACTTCAAAT |
